# Feed Additives for Coccidiosis Prevention: Comparative Evaluation of the Efficacy of Diclazuril, Robenidine and Oregano Oil in Growing Rabbits Experimentally Infected With *Eimeria* spp.

**DOI:** 10.1111/jpn.70021

**Published:** 2025-10-27

**Authors:** Florian Lohkamp, Julia Hankel, Andreas Beineke, Christina Strube, Josef Kamphues

**Affiliations:** ^1^ Institute for Animal Nutrition University of Veterinary Medicine Hannover, Foundation Hannover Germany; ^2^ Institute for Parasitology, Centre for Infection Medicine University of Veterinary Medicine Hannover, Foundation Hannover Germany; ^3^ Department of Pathology University of Veterinary Medicine Hannover, Foundation Hannover Germany

**Keywords:** coccidiosis prevention, coccidiostat, diclazuril, oregano, rabbit coccidia, robenidine

## Abstract

The present study aimed to compare the efficacy of diclazuril and robenidine, used for decades to prevent rabbit coccidiosis, with oregano oil as a potential phytogenic alternative. Four compound feed variants were tested: one variant without additive for the control group (CG), a second supplemented with diclazuril (1 mg/kg; DG), a third with robenidine hydrochloride (66 mg/kg; RG) and a fourth added with oregano oil (75 mg/kg; OG). A total of 48 SPF rabbits aged 5 weeks were kept in groups of three animals. Four groups (12 rabbits) were assigned to each of the 4 dietary variants. At Day 10 after arrival, each animal was experimentally infected with 1300 sporulated oocysts (*Eimeria media, Eimeria magna, Eimeria perforans, Eimeria flavescens* and *Eimeria coecicola*) originating from German rabbit stocks. Absolute excreted oocyst numbers were determined, *Eimeria* species identified, and reproduction rates calculated. Feed and water intake, body weight gain and feed conversion were assessed in addition to excreted faecal weights and their dry matter content. In all groups, the experimental infection resulted in markedly reduced performance parameters, changed faecal consistencies and reduced faecal weights. None of the three feed additives significantly reduced *Eimeria* reproduction or improved zootechnical parameters and faecal quality compared to the control animals. The present study confirms for the first time the complete and simultaneous ineffectiveness of diclazuril and robenidine due to multiple resistance in rabbit *Eimeria* species. Oregano as a herbal alternative for the prevention of coccidiosis in rabbits is not scientifically justified.

## 1 Introduction

Intestinal diseases are the main reason for mortality in growing rabbits and result in significant economic losses (Licois 2004; Solans et al. 2019; Lavazza et al. 2008; Schwarz et al. 2021). A common cause is coccidiosis, which is caused by unicellular, monoxenous parasites belonging to the phylum Apicomplexa and the genus *Eimeria* (Licois 2004, 2010). Coccidia are the most common parasites of the gastrointestinal tract in rabbits (Oglesbee and Lord 2020; Mäkitaipale et al. 2017) and are omnipresent on rabbit farms (Peeters et al. 1988a; Peeters and Geeroms 1992). Coccidiosis occurs on large‐scale commercial rabbit farms (Schwarz et al. 2021; Oglesbee and Lord 2020; Peeters and Geeroms 1992) as well as in small‐scale holdings, hobby breeding (Pilarczyk et al. 2020; Balicka‐Ramisz et al. 2014; Carius 2017; Basiaga et al. 2020), pet rabbits (Harcourt‐Brown 2002) and wild rabbits (Harcourt‐Brown 2002; S. M. Silva et al. 2015). Of the 11 known rabbit *Eimeria* species, 10 species infect the intestinal tract, but differ significantly in terms of their pathogenicity (Coudert et al. 1995; Pakandl 2009). *Eimeria flavescens* (*E. flavescens*) and *Eimeria intestinalis* (*E. intestinalis*) are considered highly pathogenic species, *Eimeria magna* (*E. magna*), *Eimeria media* (*E. media*), *Eimeria piriformis*, *Eimeria irresidua* as weakly to moderately pathogenic species, while *Eimeria perforans* (*E. perforans*), *Eimeria exigua*, *Eimeria vejdovskyi* are considered mildly pathogenic and *Eimeria coecicola* (*E. coecicola*) apathogenic coccidia. *Eimeria stiedai* (*E. stiedai*) is the only species that reproduces in the bile ducts and causes hepatic coccidiosis (Coudert et al. 1995).

Intestinal *Eimeria* spp. can lead to varying degrees of disease severity, with symptoms varying with the infection dose, the *Eimeria* species, the immune status and the age of the animals. Typical symptoms of intestinal coccidiosis include decreased feed intake and feed conversion, reduced faecal excretion, growth retardation, weight loss, occasional diarrhoea and mortality (Licois 2004; Harcourt‐Brown 2002; Pakandl 2009). In addition to their own pathogenicity, *Eimeria* also play a role as precursors to other, mostly bacterial secondary diseases, such as *Escherichia coli* dysentery or epizootic rabbit enteropathy (Coudert et al. 2000, 2003). The transmission of *Eimeria* spp. occurs via the faecal–oral route, with excreted oocysts requiring sporulation in the environment to obtain their infectivity.

While adult rabbits are usually asymptomatic carriers, young animals after weaning are particularly susceptible to coccidial infections (Pakandl 2009; Drouet‐Viard et al. 1997). High stocking densities (Pilarczyk et al. 2020; Duszynski and Couch 2013), litter housing (Kühn 2003; Peeters et al. 1981), moist and dirty conditions (Harcourt‐Brown 2002) favour the development of coccidiosis. *Eimeria* oocysts are remarkably persistent in the animals' environment due to their long‐lasting infectivity and their high resistance to chemical substances (Licois 2004; Pilarczyk et al. 2020; Peeters and Halen 1980). Effective hygiene management is crucial for reducing coccidial infections, but a complete interruption of the infection chain cannot be achieved under practical conditions (Licois 2004; Pakandl 2009; González‐Redondo et al. 2008). However, low‐grade infections are even desirable or necessary to induce immunity in young rabbits (Pakandl 2009; Coudert et al. 1993).

For several decades, coccidiosis prevention in both poultry and rabbits has relied on coccidiostats that are mixed into the animals' feed (Vancraeynest et al. 2008; Dorne et al. 2013; Ancillotti et al. 2024; Martins et al. 2022). Coccidiostats are feed additives that inhibit the reproduction of the parasites (Pakandl 2009; Ancillotti et al. 2024). The frequent and prolonged use of coccidiostats can lead to ineffectiveness due to chemo‐resistant *Eimeria* strains (Peek and Landman 2011; Noack et al. 2019; Chapman 1997, 2007). Drug resistance has been reported against all coccidiostats used in poultry (Peek and Landman 2011; Chapman 1997; Stephan et al. 1997). There are hardly any recent publications available on the resistance status of rabbit coccidia. According to current feed legislation in the European Union (EU) (Regulation (EC) No. 1831/2003), the only coccidiostat authorized for use in rabbits is diclazuril (European Commission 2003). Thus, rotation or shuttle programmes, as applied in poultry to reduce the development of *Eimeria* resistance (Martins et al. 2022; Chapman 2007; Abd El‐Ghany 2021; McDougald et al. 2020; Van den Ban et al. 2005), are not feasible in rabbits. The use of coccidiostats in livestock feed is controversial due to consumer concerns (Pakandl 2009; Greathead 2003; Franz et al. 2010), potential residues in food (Shao et al. 2009; Clarke et al. 2014), the environment (Ding et al. 2023; Broekaert et al. 2012; Žižek et al. 2011) and regulatory restrictions within the EU (Pakandl 2009; European Commission 2021; European Union 2018).

All these reasons have led to an increasing interest in phytogenic feed additives derived from herbs, spices, or aromatic plants (Windisch et al. 2008; Mandey and Sompie 2021; Farinacci et al. 2022), particularly as substitutes for feed antibiotics (Giannenas et al. 2016; Humer et al. 2015; Brenes and Roura 2010) and coccidiostats (Giannenas et al. 2003; Zhai et al. 2018). The Lamiaceae family, which includes oregano (*Origanum vulgare* (*O. vulgare*)), has gained special popularity due to its essential oils, which contain large amounts of the monoterpenes carvacrol and thymol (Franz et al. 2010; Windisch et al. 2008; Cuppett and Hall 1998; Burt 2004). Several studies have been published indicating that these bioactive substances exhibit anticoccidial effects in vitro (Sidiropoulou et al. 2020; Felici et al. 2023, 2020; Boyko et al. 2021; Remmal et al. 2011) in addition to their antioxidant (Giannenas et al. 2018; Botsoglou et al. 2004), antibacterial (Mitsch et al. 2004; Peñalver et al. 2005), antifungal (Conner and Beuchat 1984; Viuda‐Martos et al. 2007) and anti‐inflammatory (Braga et al. 2006; Avola et al. 2020) properties. In in vivo studies, broilers fed with an oregano oil supplement showed reduced oocyst excretion (Giannenas et al. 2004, 2003; M. A. Silva et al. 2009; Tsinas et al. 2011; Alp et al. 2012), less severe intestinal lesions (Tsinas et al. 2011) and improved feed conversion (Alp et al. 2012). On the other hand, there are also studies available indicating that oregano could not mitigate the effects of *Eimeria* infections (Scheurer et al. 2013; Arczewska‐Włosek and Świątkiewicz 2013). In fattening rabbits fed a mixture of garlic and oregano as a feed additive or receiving oregano oil via drinking water, lower oocyst excretion was observed compared to the control animals (Kowalska et al. 2012; Szabóová et al. 2012, 2021). However, this finding could not be confirmed in an own field study (Lohkamp et al. 2024).

The present study aimed to test the hypothesis that oregano oil could serve as an effective alternative to the long‐term used coccidiostats diclazuril and robenidine in preventing rabbit coccidiosis, while at the same time assessing the current efficacy of these conventional drugs against contemporary *Eimeria* field strains.

## 2 Materials and Methods

### 2.1 Animals and Housing

In the present study, 48 male, weaned, 5‐week‐old coccidia‐free (SPF) New Zealand White rabbits obtained from Charles River Laboratories (Erkrath, Germany) were used. The animals were randomly assigned to groups of three rabbits, and the feed variant groups were distributed randomly across the pens. The rabbits were housed on perforated plastic flooring with an additional elevated platform. Each pen had a base area of 7925 cm^2^, with an additional 2934 cm^2^ on the elevated platform (providing space of 2642 cm^2^ and 978 cm^2^ per rabbit, respectively), which was located 35 cm above the ground. The base area was 45% perforated, and the elevated levels had a perforation rate of 15%. The housing system complied with all requirements of German legislation (Tierschutz‐Nutztierhaltungsverordnung 2006). The light regime was set to a 12‐h day and night cycle. A 2‐h, two‐step twilight phase occurred at the beginning and end of the light phase (06:00–18:00). The experiment lasted for a duration of 42 days, like a common fattening period.

### 2.2 Experimental Infection

For the experimental infection, oocysts were extracted from rabbit droppings of two German rabbit farms. The oocysts were kept in a suspension containing 2.5% (w/w) potassium dichromate for 4 days at 26°C in a stirred glass to induce sporulation. Infection was carried out 10 days after the arrival of the rabbits (0 days post‐infection, dpi). The rabbits were inoculated *per os* with 0.2 mL of an *Eimeria* suspension applied directly under the tongue using pipette tips according to Coudert et al. (1995). Each rabbit received a dose of 1300 sporulated oocysts (750 *E. media*, 320 *E. magna*, 180 *E. flavescens*, 40 *E. coecicola* and 10 *E. perforans*).

### 2.3 Dietary Variants

In the present study, four dietary variants of one pelleted basic diet were tested. Each of these four experimental groups consisted of four animal groups, each containing three rabbits (a total of 16 animal groups, with four groups for each experimental group). One experimental group served as the control group (CG) and received the basic diet without any additives. In the experimental group DG, the compound feed was supplemented with the coccidiostat diclazuril (1 mg/kg), while the experimental group RG received the basic feed supplemented with the coccidiostat robenidine (66 mg/kg). In the fourth experimental group (OG), the basic feed was supplemented with oregano oil (*O. vulgare*, 75 mg/kg). The composition of the four feed variants is presented in Table 1.

**Table 1:** Composition of the four compound feed variants (as fed).

Component/nutrient	Diet variant (%)			
	Control	Diclazuril	Robenidine	Oregano
Alfalfa	31.3	31.3	31.3	31.3
Wheat bran	20.0	19.8	19.9	19.9
Oat hull meal	10.0	10.0	10.0	10.0
Barley	6.1	6.1	6.1	6.1
Sunflower meal	5.0	5.0	5.0	5.0
Malt culms	5.0	5.0	5.0	5.0
Wheat middlings	4.4	4.4	4.4	4.4
Grape skins	4.0	4.0	4.0	4.0
Linseed extraction meal	3.4	3.4	3.4	3.4
Corn gluten	3.0	3.0	3.0	3.0
Corn germs	2.5	2.5	2.5	2.5
Beet molasses	2.5	2.5	2.5	2.5
Vitamin and mineral premixes^a^	2.8	2.8	2.8	2.8
Diclazuril premix^b^		0.2		
Robenidine premix^c^			0.1	
Oregano oil premix^d^				0.1

^a^ Per kg feed: 14,000 IU vitamin A, 1000 IU vitamin D3, 100 mg vitamin E, 100 mg vitamin C, 0.28 mg selenium, 14.0 mg copper, 84.0 mg zinc, 42.0 mg manganese, 70.0 mg iron, 1.4 mg iodine, 0.35 mg cobalt; including the technological feed additives formic acid and lactic acid.

^b^ Per kg premix: 0.5 g diclazuril.

^c^ Per kg premix: 66 g robenidine hydrochloride.

^d^ Per kg premix: 75 g natural oregano oil.

The four compound feed variants were offered in clay troughs that were refilled twice daily. During the first 4 days, the compound feed was provided restrictively (each animal group: 100 g at −10 and −9 dpi, 200 g at −8 dpi and 300 g at −7 dpi) to gradually acclimate the young rabbits to the diet and housing conditions after transport. From −6 dpi onwards, the feed variants were offered ad libitum. Additionally, grass hay was provided ad libitum, which had been previously heated for 4 h at 80°C (hygienization) and was supplied in hay racks twice daily. Water was supplied via nipple drinkers, along with chewing wood (spruce, 4 × 4 × 10 cm), also offered ad libitum.

The chemical composition of the compound feed variants and grass hay (Table 2) was analyzed at the Institute of Animal Nutrition of the University of Veterinary Medicine Hannover. The analyses were conducted in duplicate according to the official methods of VDLUFA (Naumann and Bassler 2012). Dry matter content was determined by drying to constant weight at 103°C. The correct incorporation of diclazuril, robenidine and oregano oil in the four pellet variants was confirmed by external feed analyses by the Lower Saxony State Office for Consumer Protection and Food Safety, Feed Institute (*Niedersächsisches Landesamt für Verbraucherschutz und Lebensmittelsicherheit, Futtermittelinstitut*), Stade, Germany and IVG Institute for Grain Processing GmbH (*IGV Institut für Getreideverarbeitung GmbH*), Nuthetal, Germany. The digestible energy in the feed variants was calculated according to Villamide et al. (2009) and was per kg (as fed) 10.5 MJ DE in CG, 10.4 MJ DE in DG, 10.3 MJ DE in RG and 10.5 MJ DE in OG.

**Table 2:** Content of analyzed nutrients (% as fed).

Nutrient	Compound feed variants				Roughage
	Control	Diclazuril	Robenidine	Oregano	Grass hay
Dry matter	91.8	91.6	91.5	91.4	91.9
Crude ash	7.64	7.77	7.69	7.75	7.28
Crude protein	16.7	16.4	16.3	16.4	10.9
Crude fat	4.23	4.21	4.21	4.23	2.14
Crude fibre	14.3	14.4	14.6	13.9	27.9
NfE	49.0	48.8	48.7	49.1	43.7
Starch	9.91	9.62	9.71	9.99	—
Sugar	5.74	5.98	5.83	6.11	8.5
aNDFom	36.3	36.6	37.0	35.4	58.8
ADFom	18.5	18.2	18.7	17.7	31.5
Calcium	1.19	1.19	1.21	1.21	0.4
Phosphorus	0.65	0.65	0.65	0.65	0.3

Abbreviations: ADFom, acid detergent fibre expressed exclusive of residual ash; aNDFom, neutral detergent fiber assayed with a heat stable amylase and expressed exclusive of residual ash; NfE, nitrogen‐free extract.

### 2.4 Samples and Parameters

#### 2.4.1 Faecal Samples

**Figure 1:**
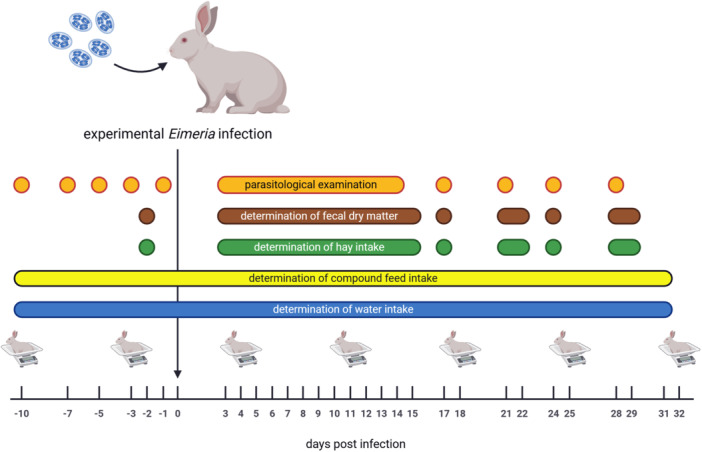
Schematic representation of the experimental procedure and data acquisition on the individual days of the experiment (created with BioRender.com).

The faeces excreted by each rabbit group were completely collected in plastic boxes placed beneath the perforated floor and emptied and cleaned daily. On 5 days before experimental infection (−10, −7, −5, −3 and −1 dpi), 20 g of faeces from each animal group were examined using a flotation method with saturated sodium chloride solution (specific gravity: 1.2) to confirm the absence of *Eimeria* spp. and other parasites. Faeces were collected on a total of 16 days post‐infection (3–14, 17, 21, 24 and 28 dpi) for parasitological examinations (Figure 1). For this purpose, the entire 24‐h faecal output from each group was hand‐sorted from the remaining hay. The faeces from each group representing three individuals were then weighed, mixed and 90% of the sample was placed in a plastic bag to determine the number of oocysts per gram of faeces (OPG), using a modified method of Coudert et al. (1995). The method was modified by adjusting the volumes to maintain the dilution factors, as instead of a 300 g sample collected over 3–4 days, 90% of the total faecal output from each individual sampling day was homogenized and used for OPG determination. Saturated sodium chloride solution (specific gravity: 1.2) was used as the flotation solution. Based on the OPG values, the total daily oocyst output (absolute number) per rabbit in each animal group was calculated as follows:

$$\mathrm{oocyst}\;\mathrm{output}\;\mathrm{per}\;\mathrm{rabbit}=\frac{\left(\frac{\mathrm{oocysts}}{g}\right)\times g\;\mathrm{total}\;\mathrm{fecal}\;\mathrm{output}\;\mathrm{of}\;a\;\mathrm{group}}{3}.$$

For each sample, 500 g of the homogenized faecal suspension from the OPG determination was kept as a reserve sample. Of this, 50 g portions were stored at 26°C in an incubator in Erlenmeyer flasks (250 mL) on magnetic stirring plates with the addition of potassium dichromate (2.5% w/w) to induce sporulation as described by Coudert et al. (1995). Subsequently, a flotation procedure using a saturated sodium chloride solution (specific gravity: 1.2) was conducted, and the *Eimeria* species were identified based on morphological characteristics as described by Eckert et al. (1995). In the faecal samples collected from 4 to 14 dpi, 1000 oocysts were identified in each sample, while in the samples from 17 to 28 dpi, 500 oocysts were identified (400× magnification, or 1000× magnification if necessary). The absolute number of each *Eimeria* species was calculated based on its percentage of the total daily oocyst output. The absolute daily excretions of each *Eimeria* species during the first 4 days of their patent period were summed to facilitate valid interpretations regarding the reproductive performance of the inoculated *Eimeria* spp. as described by Coudert et al. (1995). Additionally, the oocyst excretion in each animal group was determined over the entire trial period, that is, including reinfections.

#### 2.4.2 Zootechnical Parameters

The amount of compound feed and drinking water consumed was determined daily for each animal group by the difference between weighed‐in and weighed‐out quantities. On the days when faecal samples were collected for parasitological examinations, as well as at Days −2, 15, 22 and 29 dpi, the amounts of hay consumed were assessed for each group. For this purpose, the remaining hay in the hay racks, the pen and the hay separated from the faeces were combined, weighed and the dry matter content was determined. Hay intake was calculated by the difference between the amount provided and the amount left over based on the dry matter to avoid any misrepresentation caused by liquid (urine) adhering to the leftover hay. Body weights were recorded by weighing each rabbit individually once a week (the rabbits were tattooed), and body weight gains were calculated. Feed conversion ratio (FCR) was also determined.

At −2, 3–15, 17, 21, 22, 24, 28 and 29 dpi, the total faecal output was recorded. On the days of measuring faecal *Eimeria* excretion, the remaining 10% of droppings were used for dry matter (DM) determination. At −2, 15, 22 and 29 dpi, the total faecal output of each animal group was used for DM determination, which was measured by the weight before and after drying to constant weight (103°C). The chronological sequence of sample and data collection is shown in Figure 1.

#### 2.4.3 Clinical Parameters

The health status of the animals was recorded daily by inspection. Clinical signs of disease were documented (Table 3) and mortality was recorded.

**Table 3:** Clinical signs that were documented in the event of abnormalities.

Clinical sign
Apathy or reduced activity
Inappetence/refusal of feed
Body posture (hunched, crouched, fluffed‐up fur)
Changes in faecal consistency, soft faeces or diarrhoea
Dehydration (skin fold test)
Signs of anaemia (pale mucous membranes)
Dyspnoea, increased respiratory rate
Nasal discharge

### 2.5 Statistical Analysis

A statistical analysis was carried out using SAS Enterprise Guide (version 7.1). The normally distributed data were tested for significant differences with a one‐way analysis of variance (ANOVA) for independent samples and subsequent multiple pairwise comparisons using a pairwise *t*‐test. The non‐normally distributed data were tested with the Kruskal–Wallis rank sum test. Following the Kruskal–Wallis test, Dunn's test was performed for multiple pairwise comparisons. Time comparisons were performed separately by group with a grouped one‐way ANOVA for paired samples (repeated measurements). *p* values were adjusted by the Tukey–Kramer method. A *p* value ≤ 0.05 indicated significance for all statistical methods.

To evaluate the effect of feed on weight gain, we applied a linear mixed‐effects model using the lme4 package in R. Feed type was included as a fixed effect, while the box was specified as a random intercept to account for the clustering of animals within boxes (three animals per box). Model residuals were checked to confirm distributional assumptions. The significance of fixed effects was assessed using an ANOVA based on the fitted mixed model.

## 3 Results

### 3.1 Clinical Observations

For 13 days after the trial started, the floors of the housing facilities were totally clean and the fur on the rabbits' foot pads were completely white without any soiling. This changed abruptly at 4 dpi, when mushy, greasy faeces were found in all groups. In DG and RG, there was one rabbit each with liquid diarrhoea. The floors and foot pads of the rabbits were heavily soiled. However, the majority of the faeces at 4 dpi was formed and not conspicuous. From 5 to 8 dpi, only normal faeces were found, whereas at 9 dpi, a small proportion of faeces in all groups had a mushy consistency, but no liquid diarrhoea occurred in any rabbit group. All rabbits recovered and there were no losses during the entire trial period.

### 3.2 Oocyst Excretion

All faecal examinations conducted prior to the experimental infection confirmed that the SPF rabbits were *Eimeria*‐free. Similarly, no oocysts were found in any group at 3 dpi, while at 4 dpi, oocysts were detected in the faeces of all rabbit groups. The mean oocyst excretions of the four experimental groups on all examination days are presented as absolute values in Table 4. No significant differences were observed between the four groups on any of the examination days.

**Table 4:** Mean oocyst excretion per rabbit (±standard deviation) on each examination day and mean total oocyst excretion (±standard deviation) across all sampling days in the four groups fed the different diet variants.

dpi	Diet variant group				*p*
	Control (*n* = 4)	Diclazuril (*n* = 4)	Robenidine (*n* = 4)	Oregano (*n* = 4)	
Daily oocyst excretion (×10^6^)					
4	36.2^B^ ± 12.4	34.4^B,C^ ± 9.03	25.7^B,D^ ± 5.58	23.5^C,E^ ± 15.8	0.345
5	67.3^C^ ± 4.07	63.5^D^ ± 16.4	64.7^C^ ± 4.72	86.6^D^ ± 17.6	0.070
6	24.9^B,D^ ± 2.19	18.5^C,E^ ± 4.96	25.4^B,D^ ± 8.91	26.5^C^ ± 4.39	0.238
7	86.4^A^ ± 8.82	85.5^A^ ± 13.2	92.2^A^ ± 15.5	105^A,D^ ± 16	0.203
8	91.3^A^ ± 16.4	73.6^A,D^ ± 5.88	81.7^A^ ± 11.5	86.6^B,D^ ± 12.2	0.246
9	40.2^B^ ± 9.00	36.0^B^ ± 12.1	40.5^B^ ± 9.86	35.9^B,C^ ± 8.11	0.854
10	17.8^D,E^ ± 6.64	14.1^E^ ± 3.46	18.4^D,E^ ± 7.36	20.5^C,F^ ± 4.29	0.468
11	13.7^D,E^ ± 9.07	9.85^E^ ± 2.92	14.3^D,E^ ± 7.06	15.0^C,F^ ± 5.17	0.686
12	1.89^E^ ± 0.901	1.16^E,F^ ± 0.449	2.47^E,F^ ± 1.25	2.40^E,F^ ± 1.31	0.304
13	0.516^E^ ± 0.461	0.397^E,F^ ± 0.354	0.300^F^ ± 0.108	0.463^F^ ± 0.281	0.807
14	0.532^E^ ± 0.733	0.233^E,F^ ± 0.284	0.308^F^ ± 0.376	0.609^F^ ± 0.414	0.382
17	0.413^E^ ± 0.290	1.84^E,F^ ± 3.33	1.09^F^ ± 1.15	1.21^F^ ± 1.00	0.718
21	1.87^E^ ± 3.47	0.268^E,F^ ± 0.351	0.173^F^ ± 0.232	0.314^F^ ± 0.320	0.962
24	0.268^E^ ± 0.351	0.091^E,F^ ± 0.117	0.027^F^ ± 0.023	0.064^F^ ± 0.064	0.531
28	0.574^E^ ± 0.863	0.014^E,F^ ± 0.017	0.077^F^ ± 0.098	0.046^F^ ± 0.061	0.603
*p*	< 0.0001	< 0.0001	< 0.0001	< 0.0001	
Total	384 ± 28.9	339 ± 55.9	367 ± 37.5	405 ± 29.3	0.172
Relation	100.0	88.4	95.7	105.5	

Abbreviation: dpi, days post‐infection.

^A,B,C,D,E,F^Values differ significantly within a column (*p* ≤ 0.05).

The first *Eimeria* oocysts in the faeces at 4 dpi were exclusively *E. media*. The first *E. perforans* oocysts appeared at 5 dpi, and the first *E. magna* oocysts were identified at 6 dpi. The first *E. flavescens* and *E. coecicola* oocysts were detected at 9 dpi. The number of oocysts excreted on the first 4 patent days of each species are shown in Table 5. The differences between the four diet variant groups were not statistically significant.

**Table 5:** Mean total oocyst excretion per rabbit at the first 4 days of the patent period of each *Eimeria* species (±standard deviation) for each diet variant group.

*Eimeria* species	Diet variant group				*p*
	Control (*n* = 4)	Diclazuril (*n* = 4)	Robenidine (*n* = 4)	Oregano (*n* = 4)	
Excreted oocysts (×10^6^) *relation to control*					
*E. magna*	186 ± 18.1	164 ± 24.5	182 ± 22.3	196 ± 10.9	0.199
	*100*	*88.2*	*97.7*	*105.1*	
*E. media*	132 ± 8.92	120 ± 20.7	120 ± 3.79	142 ± 9.21	0.073
	*100*	*91.0*	*90.5*	*107.4*	
*E. perforans*	3.53 ± 0.65	3.17 ± 0.44	3.15 ± 0.36	3.46 ± 0.49	0.609
	*100*	*89.7*	*89.2*	*98,0*	
*E. flavescens*	33.1 ± 11.3	32.7 ± 12.2	31.6 ± 9.18	35.3 ± 7.47	0.964
	*100*	*98.8*	*95.4*	*106.5*	
*E. coecicola*	1.25 ± 0.35	1.07 ± 0.29	1.19 ± 0.32	1.21 ± 0.34	0.771
	*100*	*85.7*	*95.3*	*97.0*	

*Note:* Relations were calculated by setting oocyst excretion in the control group to 100%.

### 3.3 Feed and Water Intake

The compound feed and water intakes are shown in Table 6. Both parameters did not differ significantly between the groups, neither in the individual trial weeks nor over the entire trial period. In the first and third week of the trial, significantly less compound feed was consumed than in the second week of the trial.

**Table 6:** Mean daily intake of compound feed and water per rabbit and specified period in the four different diet variant groups (mean ± standard deviation).

Week	dpi	Diet variant group				*p*
		Control	Diclazuril	Robenidine	Oregano	
Daily compound feed intake per rabbit (g DM, *n* = 4)						
1	−10 to −4	69.5^B^ ± 6.55	70.1^B^ ± 4.63	72.7^B^ ± 2.62	71.4^B^ ± 1.60	0.721
2	−3 to 3	107^C^ ± 3.24	107^C^ ± 6.35	110^C^ ± 6.04	101^C^ ± 6.04	0.233
3	4 to 10	80.6^B^ ± 4.38	84.2^B^ ± 3.12	78.4^B^ ± 6.83	77.0^B^ ± 2.63	0.185
4	11 to 17	136^A^ ± 3.73	131^A^ ± 14.9	134^A^ ± 7.44	134^A^ ± 6.01	0.978
5	18 to 24	143^A^ ± 6.80	138^A^ ± 15.1	141^A^ ± 7.93	140^A^ ± 8.46	0.905
6	25 to 31	137^A^ ± 8.94	137^A^ ± 15.4	146^A^ ± 9.86	141^A^ ± 4.51	0.618
*p*		< 0.0001	< 0.0001	< 0.0001	< 0.0001	
1–6	−10 to 31	112 ± 1.84	111 ± 9.02	114 ± 5.90	111 ± 1.64	0.882
Daily water intake per rabbit (g, *n* = 4)						
1	−10 to −4	217^C^ ± 33.8	209^B^ ± 42.8	217^B^ ± 11.4	209^B^ ± 32.9	0.973
2	−3 to 3	255^B,C^ ± 33.5	246^A,B^ ± 34.1	250^B,C^ ± 16.7	254^B^ ± 36.7	0.976
3	4 to 10	253^B,C^ ± 55.9	249^A,B^ ± 42.6	225^B,C^ ± 18.8	243^B^ ± 18.8	0.726
4	11 to 17	299^A,B^ ± 34.3	312^A^ ± 64.5	324^A^ ± 23.2	310^A^ ± 48.0	0.890
5	18 to 24	315^A^ ± 27.1	309^A^ ± 58.1	311^A^ ± 18.5	310^A^ ± 48.1	0.996
6	25 to 31	289^A,B^ ± 34.1	301^A^ ± 41.9	307^A,C^ ± 28.2	300^A^ ± 31.5	0.890
*p*		0.004	0.0009	< 0.0001	< 0.0001	
1–6	−10 to 31	271 ± 30.3	271 ± 39.8	272 ± 14.8	271 ± 31.1	

Abbreviations: dpi, days post‐infection; DM, dry matter.

^A,B,C^Values differ significantly within a column (*p* ≤ 0.05).

**Figure 2:**
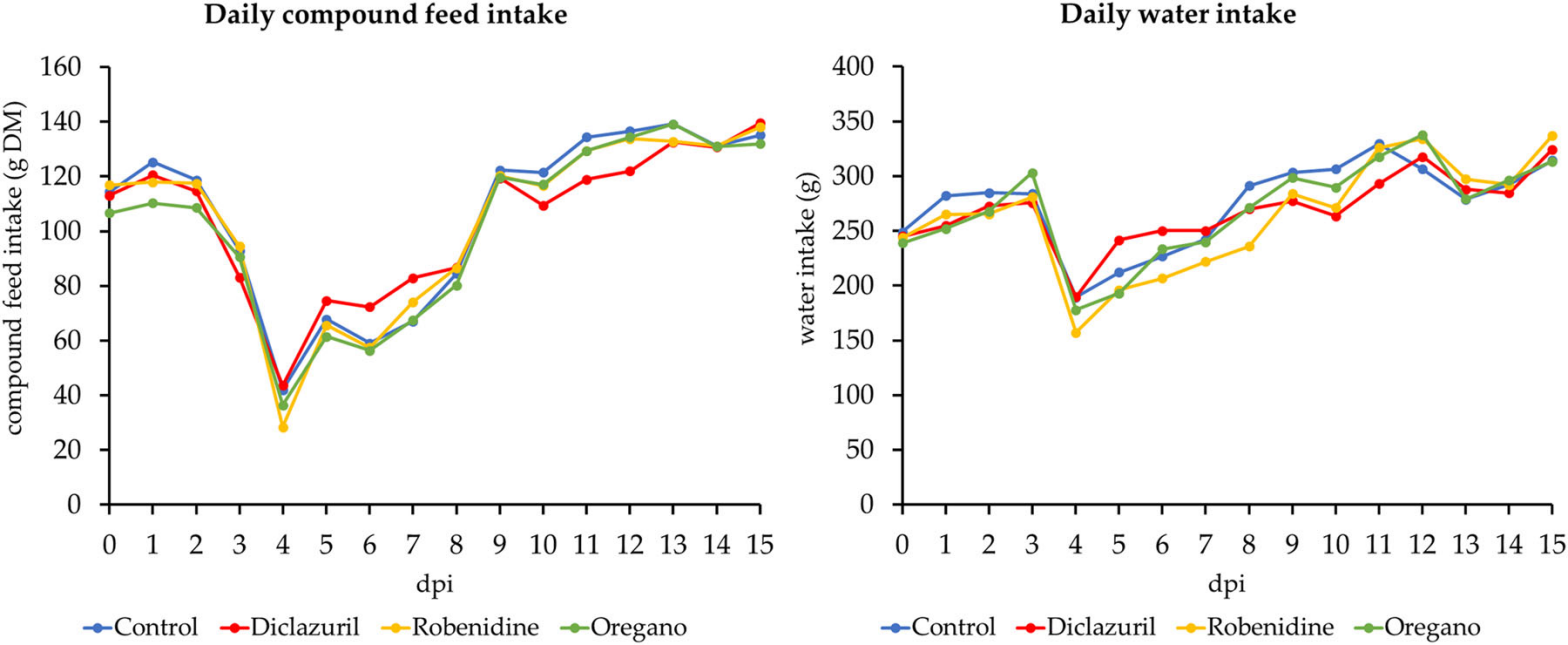
Compound feed intake (g DM) and water intake (g) per rabbit in the four different dietary variants on the first 15 days post‐infection. Standard deviations have been excluded for a clearer view.

At 3 dpi, reduced compound feed intake was recorded for the first time in all groups compared to 0 dpi, the day of infection. The lowest compound feed intake was recorded at 4 dpi (see Figure 2). At 9 dpi, they reached the level of 0 dpi again. No significant differences were found in water consumption between the groups. Water consumption was reduced for the first time at 4 dpi (Figure 2). Corresponding to the compound feed intake, the lowest water intake after experimental infection was recorded on this day. Overall, the rabbits reduced their water intake in smaller proportions than the feed intake.

Hay intake remained relatively constant in all four groups and averaged over all 20 days on which hay intake was recorded per rabbit and day in g DM: CG: 11.8 ± 3.6, DG: 13.1 ± 3.3, RG: 13.5 ± 2.9, OG: 13.9 ± 4.0. The proportion of daily hay intake to total daily DM intake from the feed averaged 9.3% in CG, 10.4% in DG, 10.6% in RG and 11.0% in OG. The highest hay intakes were recorded at 3 dpi (in g DM: CG: 14.3 ± 7.0, DG: 17.0 ± 3.5, RG: 17.3 ± 3.1, OG: 18.3 ± 3.7) and 4 dpi (in g DM: CG: 14.2 ± 4.8, DG: 16.8 ± 3.5, RG: 16.3 ± 6.7, OG: 15.8 ± 4.1). There were no statistically significant differences between the four experimental groups at any time.

### 3.4 Body Weight Gain and Feed Conversion Rate

In the third week of the trial, there was a significant decrease in body weight gains in all groups, but growth significantly increased again in the fourth week (Table 7). There were no significant differences between the four experimental groups at any time. Feed conversion increased considerably in the third week compared to the second week of the trial and decreased again in the fourth week of the trial. Significant differences between the diet variant groups could not be determined (Table 7). The mixed‐effects model indicated that variation among boxes contributed modestly to the overall variance in weight gain from Weeks 1 to 6 (random effect variance = 4.78, SD = 2.19), compared with a larger residual variance at the individual level (14.89, SD = 3.86). Estimated mean weight gain for the reference feed group was 34.4 g, and differences between feeds were minimal (–0.2 g to –0.44 g). The ANOVA revealed no significant effect of feed type on weight gain (*F*(3, 44) = 0.02, *p* > 0.99). Thus, feed treatments did not differ in their effect on growth, and box location accounted for only minor random variation without a systematic influence on the outcome.

**Table 7:** Daily body weight gain and feed conversion ratio in the four different diet variant groups (mean ± standard deviation).

Week	dpi	Diet variant group				*p*
		Control	Diclazuril	Robenidine	Oregano	
Daily body weight gain (g/d, *n* = 12)						
1	−10 to −4	37.0^A,C^ ± 7.36	40.1^A,B^ ± 8.55	37.8^A^ ± 4.48	35.8^A^ ± 7.83	0.525
2	−3 to 3	43.9^A^ ± 7.92	37.2^A,B^ ± 6.06	38.0^A^ ± 5.35	38.5^A^ ± 5.95	0.099
3	4 to 10	20.4^B^ ± 7.62	22.4^C^ ± 12.0	21.1^C^ ± 5.53	22.2^B^ ± 4.80	0.366
4	11 to 17	39.8^A^ ± 4.61	40.4^A^ ± 4.77	38.4^A^ ± 5.99	39.1^A^ ± 7.87	0.865
5	18 to 24	37.3^A,D^ ± 6.73	37.1^A,B^ ± 7.89	38.5^A^ ± 4.18	38.1^A^ ± 7.07	0.960
6	25 to 31	27.8^B,C,D^ ± 9.91	27.1^B,C^ ± 9.98	29.8^B^ ± 4.88	31.4^A^ ± 6.08	0.551
*p*		< 0.0001	0.0002	< 0.0001	< 0.0001	
Mean		34.4 ± 3.60	34.0 ± 5.65	33.9 ± 3.45	34.2 ± 4.29	0.934
Feed conversion ratio (g/g, *n* = 4)						
1	−10 to −4	2.05^C^ ± 0.159	1.95^D^ ± 0.327	2.11^D^ ± 0.137	2.21^D^ ± 0.248	0.480
2	−3 to 3	2.67^B,C^ ± 0.155	3.16^B,C,D^ ± 0.423	3.17^C^ ± 0.197	2.89^C^ ± 0.343	0.102
3	4 to 10	4.64^A,B^ ± 1.598	4.41^A,C^ ± 1.419	4.19^B^ ± 0.721	3.83^B^ ± 0.454	0.779
4	11 to 17	3.74^A,B,C^ ± 0.284	3.54^B,C,D^ ± 0.206	3.85^B,C^ ± 0.313	3.80^B^ ± 0.400	0.545
5	18 to 24	4.26^A,B^ ± 0.644	4.09^A,B^ ± 0.296	4.01^B^ ± 0.199	4.07^B^ ± 0.478	0.870
6	25 to 31	5.74^A^ ± 1.503	5.73^A^ ± 1.076	5.36^A^ ± 0.368	4.94^A^ ± 0.308	0.613
*p*		0.0004	< 0.0001	< 0.0001	< 0.0001	
Mean		3.57 ± 0.226	3.58 ± 0.189	3.67 ± 0.146	3.56 ± 0.227	0.852

Abbreviations: dpi, days post‐infection; d, day.

^A,B,C,D^Values differ significantly within a column (*p* ≤ 0.05).

### 3.5 Faecal Excretion and Faecal Dry Matter Content

Overall, the faecal masses increased over the entire course of the trial. On average over the 20 measured days, the excreted dry matter in the four groups per rabbit was 56.1 ± 14.1 g in CG; 56.4 ± 13.4 g in DG; 62.6 ± 17.6 g in RG and 56.6 ± 15.6 g in OG. These differences were not statistically significant. From 3 to 8 dpi, reduced amounts of faecal dry matter were excreted in all experimental groups compared to the examination day before inoculation (−2 dpi). The only significant difference between the groups was found at 7 dpi, when the rabbits excreted significantly more dry matter in DG than in CG (44.06 ± 1.92 g compared to 34.41 ± 4.89 g, *p* = 0.0366). The average dry matter content of the rabbits' faeces was 57.0% ± 9.07% in CG, 56.4% ± 9.11% in DG, 55.0% ± 7.92% in RG and 53.8% ± 9.62% in OG. There were no significant differences between the experimental groups on any day of the study.

## 4 Discussion

### 4.1 Clinical Symptoms and Mortality

The reduced feed intake observed in this study, along with the associated reductions in weight gain, faecal output and changes in faecal consistency, indicate that the experimental infection led to typical coccidiosis symptoms in the rabbits. All these symptoms have also been described in other studies involving *Eimeria* challenge infections (Coudert et al. 1995; Gregory and Catchpole 1986). Thus, the pathogenicity of the inoculum used in the experiment was confirmed. Coudert et al. (1995) reported that, depending on the *Eimeria* species and amount of oocysts inoculated, faeces may be generally dry during an experimental challenge, but short periods of faeces with increased fluid content or liquid diarrhoea may occur, which was also observed in the present study. The selection and composition of *Eimeria* species and the infection dose bear certain risks, as pathogenicity can vary within an certain species depending on the strain (Coudert et al. 1995). The thin line between inducing symptoms and avoiding losses to maintain equal group sizes for optimal comparability was an objective of this study, which was realized with great success.

### 4.2 Feed and Water Intake

When considering compound feed intake alone, the values for Weeks 2, 4, 5 and 6 were slightly below, and for Weeks 1 and 3 significantly below the values reported for hybrid lines of fattening rabbits of the same age (Gidenne and Lebas 1987; Gidenne et al. 2020; Maertens 2020). However, it should be noted that the values in the literature were obtained under conditions of exclusive concentrate feeding, that is, without offering hay. The provision of hay in this study may explain the reduced compound feed intake. This has also been observed in other studies, where the additional provision of roughage resulted in reduced concentrate consumption in growing rabbits compared to exclusive feeding of pellets (Capra et al. [Link], 2013; Szendrő et al. 2015). Including the hay DM intake, the total DM intake from the feed in the present study is comparable to literature values for fattening rabbits (Gidenne et al. 2020; Maertens 2020), with the exception of the first and third week. This reflects on the one hand the careful feeding regime during the first days after the young rabbits' arrival, while the reduced feed intake at 3–8 dpi is to be considered as a consequence of the *Eimeria* infection. A decrease in feed intake is a typical symptom following an (induced) *Eimeria* infection (Coudert et al. 1995; Peeters and Geeroms 1986). Several earlier studies showed that diclazuril and robenidine could prevent or at least mitigate the decline in feed intake after *Eimeria* infection (Vancraeynest et al. 2008; Coudert 1979). Such effects were not observed for any of the three additives, which could be due to ineffectiveness of the two coccidiostats as a result of anticoccidial resistance.

As expected, with the exception of the third week, the intake of concentrate increased continuously over the trial period, in line with the age of the rabbits. In contrast, the daily hay intake remained relatively constant over the trial. Available data on hay intake when compound feed is offered ad libitum to young rabbits is scarce. In a few studies in which rabbits were offered loose hay or other roughage in parallel with compound feed, the amount of roughage consumed was not measured, sometimes due to the high effort required (Capra et al. [Link], 2013; Szendrő et al. 2015; Mirabito et al. 2000). It is remarkable that the rabbits reduced their compound feed intake for several days as a result of the experimental infection, but they did not reduce their hay intake. To the authors' knowledge, there are no similar observations in the literature, probably because there are hardly any studies on rabbit coccidia in which the rabbits were offered hay.

*Eimeria* infection can lead to reduced water intake in rabbits between the 4th and 11th day after inoculation (Coudert et al. 1995), which was also observed in the present study. None of the feed additives used significantly influenced the water intake compared to the control, that is, attenuated its reduction as a consequence of the *Eimeria* infection. Overall, water consumption corresponded to that described for growing rabbits (Prud'Hon et al. 1975; Eberhart 1980; Boisot et al. 2005). In the literature, the water intake of rabbits in common feeding practice is 2–3 mL/g DM (Kamphues 2024). It is to some extent linked to the dry matter intake, but it varies depending on the feed used (Wenger 1997; Coenen 1999). For adult New Zealand rabbits, 2.19 mL water/g DM intake was determined with compound feed alone and 4.10 mL water/g DM with hay alone (Zumbrock 2002). Further studies with dwarf rabbits confirm that feeding hay alone (Wenger 1997) or a combination of hay and an energy‐rich compound feed (Prebble and Meredith 2014) leads to higher water intake compared to the exclusive supply of compound feed. Thus, the hay offered in the present study could have led to slightly higher water intake than if only compound feed had been offered.

### 4.3 Body Weight Gains

The non‐significant differences in body weight gains between the groups contradict many earlier studies in which a compound feed supplemented with robenidine (Vancraeynest et al. 2008; Coudert 1979; Peeters et al. 1979, 1980) or diclazuril (Vancraeynest et al. 2008; Vanparijs et al. 1989a, 1989b; Coudert 1990; Vereecken et al. 2012) avoided reduced body weight gain due to *Eimeria* infections compared to non‐supplemented control animals. In contrast, but in smaller numbers, there are studies in which the feed supplement robenidine (Vanparijs et al. 1989b; Vereecken et al. 2012) or diclazuril (Lohkamp et al. 2024) had no significant influence on weight gain or FCR. In a few studies, oregano leaves or an oregano extract feed supplement led to significantly higher weight gain compared to the non‐supplemented control group (Omer et al. 2013; Cardinali et al. 2015). In other investigations, however, no effects of oregano oil on performance parameters of fattening rabbits were detected (Botsoglou et al. 2004; Lohkamp et al. 2024; Soultos et al. 2009; García et al. 2014). The dietary supply of loose roughage can reduce body weight gain in growing rabbits (Szendrő et al. 2015), which may also have been the case in the present study.

The measurement of body weight development is a simple but most reliable criterion for assessing the health status of young rabbits upon a coccidial infection (Pakandl 2009; Coudert 1990; Licois 2009). With regard to this, it can be stated that none of the three tested feed additives positively influenced the course of coccidiosis and thus the health status, as no significant differences were found in comparison to the control animals.

### 4.4 Faecal Weight and Faecal Dry Matter

As in other mammals, the feed used in rabbits has a considerable influence on the excreted faecal weight (Meredith and Prebble 2017). Again, no effects of the additives tested were observed regarding this parameter. Along with the reduction in feed intake, the reduced faecal weight determined is also to be considered as a consequence of the *Eimeria* infection (Coudert et al. 1995). Furthermore, none of the three additives had a significant effect on the dry matter content in the faeces of young rabbits after experimental *Eimeria* infection. The changes in faecal quality recorded visually on 2 days were not reflected in the measured dry matter content. Presumably, the proportions of the changed faecal consistencies in the total faecal output were too low to have a decisive influence on dry matter content. Gidenne and Lebas (1987) reported that in young rabbits after weaning, initially higher dry matter values of around 80% dry matter can be observed, which, however, stabilize at around 60% dry matter after 9 weeks of life. Lower DM contents were reported by Zumbrock (Zumbrock 2002) who found DM contents of 46.6%–48.9% in the hard faeces of rabbits of different breeds fed a complete pelleted diet. In the present study, the values range between these two cited studies. It must be considered that the time‐intensive separation of faeces and residual hay increased the residence time during which the faeces were exposed to dry air in the barn and could therefore have influenced the dry matter content.

### 4.5 Oocyst Excretion

As expected, the different prepatent periods of the different *Eimeria* species explain the different daily total oocyst excretions at 4–14 dpi. It is a well‐known phenomenon in coccidia research that even under identical experimental conditions the excretion of *Eimeria* oocysts often varies considerably (Ryley and Robinson 1976). This could be an explanation for the relatively high standard deviations observed in the number of excreted oocysts in the groups. Reinfections, which occurred to varying degrees in the animal groups, may be responsible for the increasing variation in oocyst numbers between the groups as the experiment progressed. The results of the *Eimeria* species identification in the present study confirm the prepatent periods reported by Coudert et al. (1995).

The *Eimeria* reproduction results, particularly for diclazuril and robenidine, contradict many earlier studies. Both in experimental *Eimeria* infections under laboratory conditions as well as in naturally infected rabbits in field studies, high efficacies, that is, reduced oocyst excretion in some cases by more than 99% or even complete inhibition of *Eimeria* reproduction were determined when the compound feed was supplemented with robenidine (Peeters and Halen 1980; Vancraeynest et al. 2008; Peeters et al. 1979; Maertens et al. 2000; Maertens and Van Gaver 2010) or diclazuril (Vanparijs et al. 1989a, 1989b; Coudert 1990). Additionally, these substances significantly improved body weight gain and feed conversion compared to non‐supplemented control animals (Peeters et al. 1979, 1980; Vanparijs et al. 1989a; Vanparijs et al. 1989b; Coudert 1990; Maertens et al. 2000).

It is reported that all tested rabbit *Eimeria* spp. were completely sensitive to robenidine before it came to the market in 1982, and that this active substance led to a considerable reduction in coccidiosis on commercial rabbit farms in its first years of use (Licois 2004; Peeters and Geeroms 1992; Peeters et al. 1988b). After a few years of use, robenidine‐resistant *E. magna, E. media* and *E. perforans* were reported (Peeters et al. 1988a; Vanparijs et al. 1989b; Peeters et al. 1987) and were subsequently considered to be common in rabbitries (Licois 2004; Vereecken et al. 2012; Coudert 2004; Licois and Marlier 2008). Robenidine remained the drug of choice due to its good efficacy against the two highly pathogenic species, *E. intestinalis* and *E. flavescens* (Licois 2004, 2010; Coudert 2004; Licois and Marlier 2008). The present study fundamentally undermines this long‐standing assumption. A recently published field study provides indications for diclazuril‐resistant *Eimeria* species from a German rabbit farm (Lohkamp et al. 2024). The present study draws the conclusion, for the first time, that all rabbit *Eimeria* species tested here were resistant to both substances. In view of the fact that only diclazuril and robenidine have been used in rabbit husbandry for decades, the resistance of rabbit *Eimeria* spp. to these substances is not surprising.

By fully measuring the excreted faecal masses, oocyst shedding was determined as absolute values, which is significantly more accurate and meaningful than expressing it as OPG. The daily faecal collections in the ‘important phase’ after infection (3–14 dpi) made it possible to determine the reproduction of all five inoculated *Eimeria* species, namely on their first 4 patent days. According to the *Guidelines on Techniques in Coccidiosis Research* (Coudert et al. 1995), only these absolute oocyst excretions from each species on their first 3–4 patent days allow valid interpretations because they indeed reflect up to 99% of reproduction without being influenced by possible (and varying) reinfection. In this study, 90% of the daily excreted faecal mass from each animal group was examined parasitologically, which additionally contributes to accurate results. Only oocysts from a field isolate were used, allowing conclusions on the resistance status of *Eimeria* spp. in the field.

### 4.6 Evaluation of Oregano

In the present study, no effects of oregano oil on *Eimeria* reproduction in rabbits could be detected. This contrasts with several in vitro studies with promising anticoccidial effects of oregano oil. It must be emphasized that it is difficult to extrapolate the promising doses used in vitro to in vivo (Sidiropoulou et al. 2022) and that in vitro active substances are not necessarily active in animals (McDougald 1986). This study was also unable to confirm in vivo studies with oocyst‐reducing effects in rabbits with oregano supplementation. Szabóová et al. (2012) supplemented the drinking water of fattening rabbits with oregano oil over the first 21 days of fattening and found reduced OPG values for the oregano oil groups at Days 21 and 42, that is, even 3 weeks after the end of oregano supplementation. Similarly, rabbits fed a mixture of herbal extracts (oregano, cinnamon and Mexican pepper) in the first 3 weeks of fattening showed lower OPG values on the two examination days of the study. Szabóová et al. (2021) reported again in 2021 on a study in which oregano oil (via drinking water), the plant extract mixture and two other additives were tested. Once again, reduced OPG values were reported in rabbits (at Days 21 and 42 of the fattening period) that received oregano oil or the extract mixture, and this was again observed 3 weeks after removing these substances.

Kowalska et al. (2012) investigated five different garlic–oregano oil combinations in compound feed for fattening rabbits, with some combinations showing reduced OPG values on 2 examination days and one combination improving body weight gain during one study phase. In an own field study, no effects on OPG values or fattening performance were found in naturally *Eimeria*‐infected fattening rabbits (Lohkamp et al. 2024).

When evaluating the suitability of oregano oil as a herbal substitute for conventional coccidiostats, the present study should be considered conclusive due to the following reasons: all rabbits had the same prior exposure to coccidia, as they were immunologically identical (‘naive’) with respect to *Eimeria* spp., and all rabbits were experimentally infected individually at the same time with an identical, defined dose of oocysts containing defined, current, field‐relevant *Eimeria* species. Furthermore, all rabbits were kept under standardized housing and feeding conditions and an intensive animal monitoring was conducted, with precise measuring of compound feed, hay and water intake as well as faecal quality. The excreted faeces from each examination day were completely weighed to obtain absolute oocyst excretion values instead of faecal weight‐dependent concentrations (OPG). Instead of spot faecal examinations, daily faecal examinations were conducted during the crucial reproductive phase of *Eimeria* after infection (3–14 dpi), allowing for quantification of the reproduction of the inoculated *Eimeria* spp. without influence from potential reinfections (which may differ between groups). Detailed species identification allowed for precise excretion values for the five different *Eimeria* spp. Performance parameters, such as body weight gain and feed conversion rate, were determined in the best possible comparability, that is, without influence from other diseases or differing animal numbers due to losses.

The results of this study confirm that the intake of essential oils or extracts from various herbs and spices appears to have far fewer positive effects in rabbits than possibly in broilers and piglets, which is probably due to the specific digestive physiology of rabbits (Erdelyi et al. 2008; Dalle Zotte et al. 2016).

### 4.7 Influence of Hay Feeding on the Results

In contrast to all the studies in the past decades on diclazuril and robenidine effects mentioned above, the rabbits in the present study were offered hay. This certainly led to a dilution of the test substances in the digestive tract of the rabbits. However, the additional offer of structured roughage is nowadays considered contemporary because it is increasingly demanded in rabbit husbandry and it is already mandatory for commercial rabbit farms in Germany (Tierschutz‐Nutztierhaltungsverordnung 2006). Thus, the ad libitum supplementation of roughage to the compound feed in the present study realistically reflects the conditions in practice and complies with the law. In addition, the hay intake must be regarded as low overall compared to the compound feed intake, as it accounted for an average in the groups of around 9.3%–11% of the daily dry matter intake on the measured days. Assuming similarly high effects of the coccidiostats as in the mentioned studies from the past decades, the active substance concentrations achieved here should have shown stronger effects despite a slight dilution effect due to the hay feeding. This is because the concentrations of dicalzuril and robenidine here were above the doses that achieved good efficacy in earlier studies, both in terms of oocyst excretion and growth performance. Half the diclazuril dose used here, that is, 0.5 mg diclazuril/kg feed, reduced the reproduction of *E. flavescens* in experimentally infected rabbits by more than 95% compared to non‐treated control animals (Coudert 1990). In another study, the same dose led to a 99.1%, 99.0% and 96.3% reduction in oocyst excretion compared to non‐treated control animals at Days 10, 14 and 21 after infection with *E. flavescens, E. intestinalis, E. magna* and *E. perforans*, respectively (Vanparijs et al. 1989a). In a further study with 0.5 mg diclazuril/kg feed, the multiplication of inoculated *Eimeria* spp. (*E. intestinalis, E. magna* and *E. perforans*) was even completely inhibited as of Day 14 p.i (Vanparijs et al. 1989b). In all three publications, body weight gains were significantly improved with this concentration of active ingredient compared to the control animals without coccidiostat. Studies are also available for robenidine with similarly good effects at lower doses than that used here. In rabbits with *Eimeria* challenge infections (*E. intestinalis, E. magna, E. perforans* and *E. stiedai*) and in rabbits with natural infections (*E. flavescens, E. intestinalis, E. magna, E. media* and *E. perforans*), the addition of 33 mg robenidine/kg feed significantly reduced oocyst output and increased body weight gain (Peeters et al. 1979, 1980).

## 5 Conclusion

In the present study, neither diclazuril and robenidine, nor the phyto‐additive, oregano oil, significantly inhibited *Eimeria* spp. reproduction. In connection with this, none of the substances tested reduced coccidiosis symptoms in the experimentally infected rabbits. The present study confirms that after decades of using diclazuril and robenidine as feed additives, anticoccidial double resistance might be widespread on rabbit farms in the field, resulting in loss of efficacy of these additives. With regard to oregano oil, in immunological identical animals (all *Eimeria*‐naïve) under standardized conditions, no coccidiostatic effects could be detected, which means that its suitability as a ‘herbal coccidiostat’ is at least questionable. Based on the results of this study, the question raises whether the use of feed additives for coccidiosis prevention in rabbits is still justified or useful today, or whether other strategies such as promoting the development of *Eimeria* vaccines or breeding for higher parasitic resilience would be better alternatives.

## Ethics Statement

The authors confirm that the ethical policies of the journal, as noted on the journal's author guidelines page, have been adhered to and the appropriate ethical review committee approval has been received. The authors confirm that they have followed EU standards for the protection of animals used for scientific purposes and feed legislation. All animal procedures were carried out in accordance with ethical guidelines for the use of animal samples as approved by the Ethics Committee of Lower Saxony for the Care and Use of Laboratory Animals, LAVES (Niedersächsisches Landesamt für Verbraucherschutz und Lebensmittelsicherheit; Reference No.: 33.19‐42502‐04‐19/3261) and were carried out in accordance with the German Animal Welfare Legislation.

## Conflicts of Interest

The authors declare no conflicts of interest.

## Data Availability

The data that support the findings of this study are available from the corresponding author upon reasonable request.
